# Treating Older Children with Clubfoot: Results of a Cross-Sectional Survey of Expert Practitioners

**DOI:** 10.3390/ijerph20196846

**Published:** 2023-09-27

**Authors:** Grace Drury, Timothy R. Nunn, Firaol Dandena, Tracey Smythe, Christopher B. D. Lavy

**Affiliations:** 1 Nuffield Department of Orthopaedics Rheumatology and Musculoskeletal Sciences (NDORMS), University of Oxford, Nuffield Orthopaedic Centre, Windmill Road, Oxford OX3 7HE, UK; 2 CURE Children’s Hospital of Ethiopia, P.O. Box 21634, Addis Ababa 1000, Ethiopia; 3 International Centre for Evidence in Disability, Department of Population Health, London School of Hygiene & Tropical Medicine, London WC1E 7HT, UK; 4 Division of Physiotherapy, Department of Health and Rehabilitation Sciences, Stellenbosch University, Cape Town 7602, South Africa

**Keywords:** clubfoot, delayed presentation, walking age, neglected, immature, treatment, Ponseti, older child

## Abstract

Treating clubfoot in walking-age children is debated, despite studies showing that using the Ponseti casting principles can correct the midfoot effectively. We aimed to explore techniques and approaches for the management of older children with clubfoot and identify consensus areas. A mixed-methods cross-sectional electronic survey on delayed-presenting clubfoot (DPC) was sent to 88 clubfoot practitioners (response rate 56.8%). We collected data on decision-making, casting, imaging, orthotics, surgery, recurrence, rehabilitation, multidisciplinary care, and contextual factors. The quantitative data were analysed using descriptive statistics. The qualitative data were analysed using conventional content analysis. Many respondents reported using the Pirani score and some used the PAVER score to aid deformity severity assessment and correctability. Respondents consistently applied the Ponseti casting principles with a stepwise approach. Respondents reported economic, social, and other contextual factors that influenced the timing of the treatment, the decision to treat a bilateral deformity simultaneously, and casting intervals. Differences were seen around orthotic usage and surgical approaches, such as the use of tibialis anterior tendon transfer following full correction. In summary, the survey identified consensus areas in the overall principles of management for older children with clubfoot and the implementation of the Ponseti principles. The results indicate these principles are well recognised as a multidisciplinary approach for older children with clubfoot and can be adapted well for different geographical and healthcare contexts.

## 1. Introduction

Clubfoot, also known as congenital talipes equinovarus (CTEV), is one of the most common congenital musculoskeletal deformities. The highest birth prevalence rates are observed in low- and middle-income countries (LMICs), particularly in the South East Asia region, where it is estimated to be 1.80 (95% CI: 1.32–2.28) per 1000 live births, and the Africa region (1.31, 95% CI: 0.86–1.77). In these regions, an estimated 60,307 and 51,874 children, respectively, are born with clubfoot each year [1]. The Ponseti technique of treatment, which is primarily non-surgical, can successfully treat clubfoot in up to 95 percent of cases [2].

In the first phase of the Ponseti treatment for clubfoot in infants, foot deformity correction is performed with foot manipulation and weekly plaster cast changes. The cavus deformity is treated first, followed by the adductus deformity. When the talar head is covered, a percutaneous Achilles tendon tenotomy is usually performed to correct the ankle equinus. In the second (maintenance) phase, the corrected position of the clubfoot is maintained using a foot abduction brace (FAB) to prevent relapse of the deformity. The FAB is worn for 23/24 h for the first 12 weeks, and thereafter during sleep until the child is 4–5 years old [3].

Treatment of clubfoot is more challenging in older children and in those with greater deformity [4]. ‘Delayed-presenting clubfoot’ (DPC) refers here to clubfoot abnormalities, of idiopathic cause, present at birth and not treated before walking stage [5]. We use ‘delayed-presenting clubfoot’ (DPC) in this paper as a preferred nomenclature as the term ‘neglected clubfoot’ infers a degree of blame and deliberate inaction. DPC is common in LMICs, particularly in rural and remote places, due to a lack of access to clubfoot treatment. The rigidness of the deformity increases as a child gets older in part due to the changing viscoelastic properties of the soft tissues and in part as the bony skeleton matures. While the Ponseti method is proven to be extremely effective at managing clubfoot when used early in life, particularly in infancy, there is comparatively less published literature on the management of DPC. 

However, there are a growing number of reports of older children being successfully treated utilising the Ponseti principles of management [6,7,8]. Published studies looking at outcomes of the application of the Ponseti principles in older children have shown generally excellent correction of the midfoot deformities by following the casting sequence as recommended by Ponseti. It is clear that the process takes much longer, and the initial cavus deformity takes multiple cases to correct, but it is effective in a significant majority of idiopathic cases. Several authors have tried to review the outcomes of clubfoot treatment [8,9,10]. A common theme amongst these reviews is the recommendation for higher quality and higher volume studies to provide a greater amount of data upon which to draw solid conclusions. Obtaining such data is very challenging and it is noteworthy that the studies published typically contain patient outcome data with around three years’ follow-up. Arguably, this is insufficient data for accurate conclusions on longer-term outcomes. 

Therefore, due to the gap in detail on or consensus of how LMIC multi-disciplinary practitioners treat older children with clubfoot in the published literature, including why and how the Ponseti principles are applied, the objectives of this formative research study were to:

- explore current treatment techniques and approaches for management of older children with clubfoot, to gain insight on all aspects of care within a multidisciplinary team, including typical pitfalls;
- identify areas of management consensus as well as to define controversial aspects that would be useful for future research;
- inform the development of a new training course on the management of DPC in a resource-limited setting.

This paper uses the Standards for Reporting Qualitative Research guideline for reporting qualitative research [11].

## 2. Materials and Methods

A major barrier to the provision of clubfoot treatment in Africa is the critical shortage of healthcare providers [12] and a lack of providers’ knowledge and skills [13]. Johnson et al. (2017) conclude that there should be a global implementation of structured training programmes that promote conservative manipulative methods to manage clubfoot [13]. The context for our study was a project (referred to in this study as “the project”) to develop a new training course on treatment and surgical care for walking children with DPC in Ethiopia, funded by an award granted to the Nuffield Department of Orthopaedics, Rheumatology and Musculoskeletal Sciences (NDORMS) at the University of Oxford and to the CURE Children’s Hospital of Ethiopia in 2020 by the Africa Mother & Child Grants Programme. The development of the new DPC training course would be informed by the formative research of this study and by lessons learned from the partners’ previous collaboration on standardised courses for training healthcare providers in Africa in the treatment of infants with clubfoot using the Ponseti method.

Limited published data exist on current practices and practitioners’ rationale for DPC management approaches. We used a mixed-methods approach for this study to gain a deeper understanding of the current management of DPC that could influence the design of a new training course. We conducted an online cross-sectional self-reported survey among clubfoot practitioners, collecting both quantitative and qualitative data to provide a comprehensive context for treatment decisions. The survey included multiple-choice questions to collect measurable data on the frequency of or preferences for management options. Additionally, we used an inductive descriptive qualitative approach to investigate the clinical and contextual reasons related to decision-making processes for DPC management options. 

The multi-disciplinary study team included the project directors (C.L. and T.N., both consultant orthopaedic surgeons), project manager (G.D.), two children’s physiotherapists (T.S. and Rosalind Owen), and a research coordinator (F.D.) based at the CURE Children’s Hospital of Ethiopia. C.L., T.N., R.O. and T.S. all have direct experience of clubfoot treatment, training, and research in the UK and Africa over the past 15 years. T.N. and F.D. are based at the largest children’s orthopaedic service in Ethiopia which receives referrals from across the country and treats around 120 children with DPC per year. Some of the survey respondents were known to members of the study team through professional networks; therefore, the data were anonymised for data collection and analysis.

The study aimed to include multidisciplinary practitioners regularly treating older children with clubfoot in a LMIC setting. This encompassed surgeons, doctors, physiotherapists, and clinical officers, as clubfoot care may be provided by different cadres in settings with a scarcity of trained providers and resources. We targeted the survey invitation to experienced practitioners to increase the validity and quality of responses and to more efficiently analyse the volume of data from the detailed survey. Due to time and resource constraints, the survey was conducted in English only. We included responses from all countries where DPC is commonly seen.

The study was conducted according to the guidelines of the Declaration of Helsinki. Ethical review and approval were waived for this study, as assessed by the Oxford Tropical Research Ethics Committee protocol, University of Oxford. The data were anonymised for the purpose of data analysis. Informed consent was obtained from all subjects involved in the study.

We selected an electronic survey as a data collection tool using multiple-choice questions to collect data with measurable variables on preferred casting and surgical and rehabilitation techniques, and open-text questions to collect qualitative data on the context of clinical decision-making. The cross-sectional survey enabled us to collect consistent data over the same time frame, i.e., in parallel with the course content design.

We included questions on all phases of the multidisciplinary management of delayed-presenting clubfoot, based on data from a 2018 pilot project provided by the Global Clubfoot Initiative surveying referral pathways and rehabilitation protocols for older children with clubfoot in low-resource settings. We divided the survey into sections on decision-making, casting, the role of imaging, type of surgery and need for orthotics, rehabilitation and post-operative management, multi-disciplinary approaches, and contextual factors. 

We refined the scope of the survey to management protocols for children aged 2–10 years with congenital idiopathic clubfoot that can benefit from the principles of the Ponseti technique. The ‘Ponseti principles’ are defined here as serial casting to achieve foot correction, starting with cavus elimination, followed by adduction correction, followed by the surgical correction of equinus at the ankle. This survey excluded protocols for patients who had skeletally mature feet, had cast-resistant clubfoot following extensive correction casts, severe deformity that required osteotomies or arthrodesis, or external fixation treatment of residual deformity. Further exclusions were protocols for syndromic or neurogenic clubfoot.

We undertook a cross-sectional electronic survey between 12 October and 10 November 2020 using the JISC Online Surveys platform. C.L. and T.N. sent the survey by email invitation to 38 experienced practitioners (20 practitioners selected by the study team through professional experience, and 18 Global Clubfoot Initiative Advisory Board members). The Global Clubfoot Initiative also sent survey invitations to 50 member contacts to cascade directly to experienced practitioners worldwide. The collected survey data were held on a secure survey platform.

There were 50 survey respondents out of the 88 contacts who were sent the survey, which is a response rate of 56.8%. Forty-nine of the respondents currently treated older children with clubfoot. The one respondent who did not currently treat older children had recent clinical experience with DPC as a visiting surgeon in a low-resource setting and therefore the study team decided to include this response due to relevant experience. 

The quantitative data were analysed by F.D. and T.N. using descriptive statistics where appropriate. G.D., T.N. and F.D. collated the responses to the open-ended questions into a word document and undertook qualitative content analysis using the questions as codes. We grouped similar responses, titled these, and counted the number of responses. Using conventional content analysis, coding groups were derived directly from the text data. We analysed qualitative questions thematically and undertook a narrative description of themes raised in the electronic survey.

The survey data were summarised and presented in written format to the project’s multidisciplinary technical advisory group (composed of invited highly experienced trainers in the management of DPC in low-resource settings) and the project’s stakeholder group in December 2020. We held online meetings with these two focus groups to review common survey result themes for triangulation, asking them to verify the accuracy or resonance with their perspectives, and to determine which management aspects would be appropriate in developing the DPC course. 

## 3. Results

### 3.1. Participant Demographics

**Figure 1:**
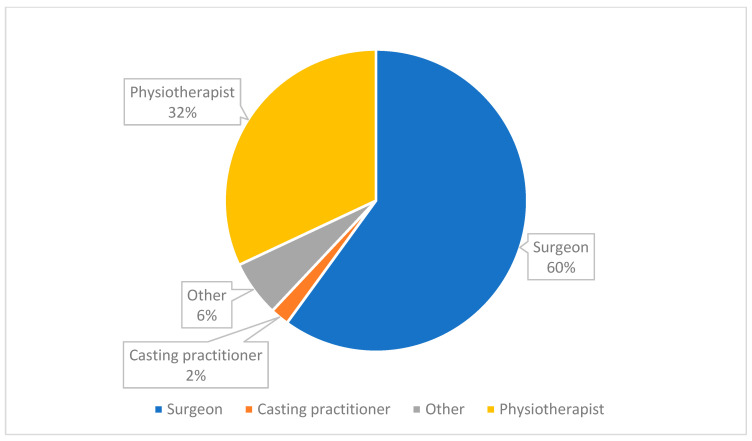
Medical cadre of survey respondents (*n* = 50).

There were thirty (60%) surgeons, sixteen (32%) physiotherapists, one (2%) casting practitioner and three (6%) ‘other’ respondents to the survey (see Figure 1).

Thirty-seven of the respondents specified their country of work (here listed in alphabetical order): Australia, Bangladesh, Cameroon, Egypt, Ethiopia, India, Indonesia, Kenya, Mali, Mexico, Mozambique, Nepal, Nicaragua, Niger, Norway, Philippines, Republic of Congo, Rwanda, Spain, Tanzania, United Kingdom, United States of America, and Zimbabwe. The remaining thirteen did not specify. The most common country of work cited was ‘Ethiopia’ (7 respondents).

**Figure 2:**
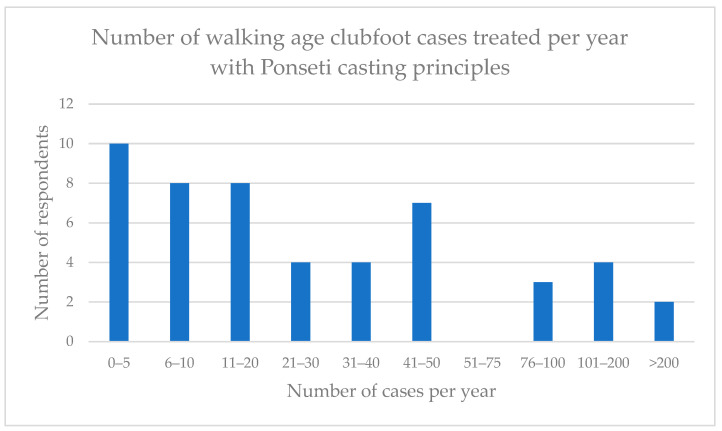
Number of walking-age clubfoot cases managed by respondents with Ponseti casting principles per year.

The number of DPC cases treated by the respondents per year was quite variable (Figure 2).

### 3.2. Decision-Making

During the assessment, most practitioners reported that they would try to evaluate the severity of the deformity and clubfoot flexibility. Practitioners also stated that they reviewed the spine, the neurological status, the tendon motor function of the foot, the standing position of the foot, and the walking pattern. The scoring systems that respondents most commonly used to assess children with DPC were the Pirani score (cited by twenty-two respondents) [14] followed by the PAVER score (cited by eight respondents) [5]. Several respondents noted that, although the Pirani score was used, it was not designed, validated, or well suited for the walking child.

Practitioners also factored in several contextual considerations when making treatment decisions. These considerations included the patients’ distance from the facility, patients’ financial situation, home environment, nutritional evaluation, damage to cast, and factors related to the patient dropping out of treatment or missing follow-up appointments. 

### 3.3. Casting

Long leg casts were favoured by a large majority of respondents, 83.7% (*n* = 41), instead of short leg casts. Most respondents, 54% (*n* = 27), gave non-weight-bearing instructions, whilst 20% (*n* = 10) recommended weight-bearing and 26% (13) said it depended, for example, on factors such as age, mobility, or family circumstances. Children who have bilateral clubfoot can have both treated at the same time and 82% (*n* = 41) of respondents would prefer this. Social factors, schooling, and the size of the child were amongst the reasons cited for sequential correction of bilateral clubfeet. 

The commonest cast change interval was weekly (48%, *n* = 24), followed by a cast change every two weeks (40%, *n* = 20). Again, the age of the child and social factors were qualifiers in the responses, with advice for younger children to receive weekly cast changes. Respondents defined the indications of a ‘successful cast’ in terms of correction of deformity, plantigrade foot, and reduction in the talar head. 

We asked respondents when they would ‘abandon casting and pronounce the foot as “cast resistant”’. There was a range of answers and no clear consensus on the number of casts attempted or maximum duration for serial casting, but, in general, practitioners were looking to see progress with deformity correction through serial casting and would stop when they did not see any further improvement.

### 3.4. Orthotics

When we asked practitioners if they recommended orthotics, 84% (*n* = 42) said ‘yes’, 12% (*n* = 6) said ‘no’, and 4% (*n* = 2) said they ‘don’t know’. Some qualified their response according to whether they believed there was going to be compliance, whether a facility was available, or whether a tendon balancing procedure was performed. Foot abduction braces (FABs) and ankle foot orthoses (AFOs), used as night splints, were common orthotic preferences. 

### 3.5. Surgical Options

**Figure 3:**
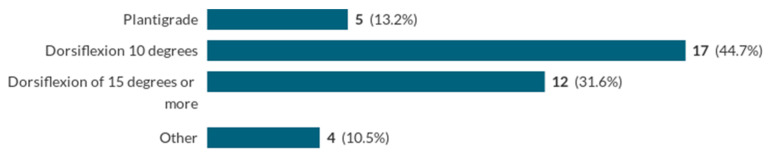
What is your surgical goal of achieving full correction of the equinus deformity?

Respondents were asked about the dorsiflexion goal of surgical correction of the equinus deformity. An ability to achieve a dorsiflexion goal of 15 degrees was seen as satisfactory (see Figure 3):

If the dorsiflexion goal was not achieved, the most common response (cited by eight practitioners) was to perform serial casting again, followed by managing the problem with osteotomies (cited by six practitioners). Posterior ankle capsular release was ‘always’ included in the protocol of 7.9% (*n* = 3) practitioners, ‘sometimes’ in that of 68.4% (*n* = 26), and ‘never’ in that of 15.8% (*n* = 6). A total of 35.1% (*n* = 13) of respondents perform cast wedging to gain further dorsiflexion and 59.5% (*n* = 22) do not do this.

The most commonly suggested indications for tibialis anterior tendon transfer following full correction (TATT) were dynamic supination (cited by four respondents) and expected low compliance with bracing in their setting (cited by three respondents). Eleven out of eighteen respondents (61.1%) would transfer the tendon in a TATT to the lateral cuneiform, with a few respondents opting for the intermediate cuneiform.

Regarding the preferred technique for Achilles tendon lengthening, 18.4% (*n* = 7) of respondents used Achilles tendon lengthening, 23.7% (*n* = 9) used tenotomy, and 57.9% (*n* = 22) of respondents used either technique.

### 3.6. Imaging

A total of 47.4% (*n* = 18) of respondents used pre-operative imaging; 52.6% (*n* = 20) did not. Of the 50% (*n* = 19) of respondents who performed tibialis anterior tendon transfer following full correction (TATT), 36.8% (*n* = 7) used intra-operative imaging whereas 63.2% (*n* = 12) do not. For clubfeet with resistant equinus, the use of lateral radiographs or intraoperative fluoroscopy was suggested as helpful. This helps by defining the cause, which could be due to a hidden midfoot cavus/plantaris deformity which can be difficult to clinically appreciate.

### 3.7. Anaesthesia

A total of 65.8% (*n* = 25) of respondents used general anaesthesia for Achilles tendon lengthening for children aged 2–10, 13.2% (*n* = 5) use local anaesthesia, and 21.0% (*n* = 8) said ‘it depends’. There was no common theme in the reasons given for different anaesthesia choices. It was unclear from the responses if there was consensus on the age threshold for the use of general anaesthesia.

### 3.8. Recurrence

A total of 82% (*n* = 41) of respondents said that they had seen recurrences of clubfoot deformities and 12% (*n* = 6) had seen over-corrections in DPC management. (Findings from a study in New Zealand similarly reported recurrence as a common problem, where twenty-one (41%) of fifty-one patients had a recurrence, which was major in twelve of them and minor in nine [15]). The most common cause of deformity recurrence (suggested by 34% (*n* = 17) of respondents) was the lack of adherence to the treatment protocol. Other factors commonly suggested were that patients were lost in follow-up or that the clubfoot may have been under-corrected. The most common management approach for DPC recurrence cases was to perform serial casting again and surgery.

### 3.9. Rehabilitation and Post-Operative Management

A total of 52% (*n* = 26) of respondents performed post-operative casting, whereas 38% (*n* = 19) did not. Of those who performed post-operative casting, both the mean and median time point post-operation for changing the cast was two weeks. In terms of post-surgery weight-bearing status in the cast, 63.2% (*n* = 24) of respondents would recommend no weight-bearing, 26.3% (*n* = 10) would recommend ‘weight bearing as tolerated’, and 10.5% (*n* = 4) would recommend partial weight-bearing.

The most common rehabilitation exercises suggested focused on strengthening and improving the flexibility of the foot and ankle. These exercises aimed to enhance the range of motion, muscle strength, and proprioception. Specific stretches included those for the calf muscles. Other exercises involved toe curls, ankle range of motion exercises, and balancing activities to improve stability. There was some focus on rehabilitation exercises for the whole child, including core and hip strength. 

### 3.10. Multi-Disciplinary Approach

Most practitioners recommended that multi-disciplinary teams (MDTs) (which may include surgeons, physiotherapists, doctors, orthotists, and social workers) be involved in the management of older children with clubfoot, with a team approach to planning, decision-making, follow-up, and rehabilitation. Respondents suggested that the most important process for enhancing a multidisciplinary approach was frequent collaborative team communication throughout the treatment pathway, extending to clear communication to parents and caregivers.

### 3.11. Summary of Results

The main quantitative results from the study are summarized in Table 1:

## 4. Discussion

The study included responses from 50 participants, including surgeons, physiotherapists, and casting practitioners from high-, middle-, and low-income countries, representing a total of over 23 countries. The information provided by the respondents highlights key findings regarding decision-making, casting, orthotics, surgical options, imaging, anaesthesia, recurrence, rehabilitation, and a multidisciplinary approach in the management of clubfoot deformity in older children. Regarding decision-making, practitioners consider various factors during assessment, indicating a comprehensive approach. However, the limitations of the Pirani score for walking children are acknowledged, suggesting the need for more suitable assessment tools. The consideration of contextual factors reflects a holistic approach to treatment planning. The majority of practitioners recommend orthotics, particularly foot abduction braces (FABs) and ankle foot orthoses (AFOs). This emphasises the view that it is important to provide external support to maintain correction and facilitate proper foot positioning. The range of surgical options available indicates a tailored approach based on individual cases, with an emphasis on achieving dorsiflexion goals and addressing specific deformities. The utilisation of pre-operative and intra-operative imaging varies among practitioners, suggesting a lack of consensus on their necessity. Anaesthesia choices for Achilles tendon lengthening vary, with general anaesthesia being the most common approach. The recurrence of clubfoot deformity and over-corrections indicate challenges in long-term management, with non-adherence to treatment protocols being a prominent factor. Serial casting and surgery are the main strategies for managing recurrence cases.

Whilst the numbers of patients presenting with DPC is small in high-income settings, the volume of cases is significant in low-income countries where healthcare provision is limited. Respondents from low-income countries had a large volume of cases and experience in this type of treatment. In combination with the prevalence of this untreated condition, the social factors for prolonged treatment and disruption to families and livelihoods are also disproportionately higher in low-income settings. Contextual factors, including social and family issues, are therefore much more relevant in these settings and are significant influences on the choice of treatment options [16]. As we saw clearly from the survey, decisions on timing of treatment start, timing of cast change, and bilateral treatment are closely related to the availability of accommodation close to the treatment facility and ability to access care throughout the serial casting, surgical, and rehabilitation phases. In terms of the assessment of the clubfoot deformity, the continued use of the Pirani score for the assessment of clubfoot in older children was a surprise to the study team, as many of the components of the score are not relevant to older children of walking age, which was also noted by several respondents. (For example, posterior creases are not seen in this population, medial plantar creases are uncommon, and differentiation of the ‘empty heel’ and different degrees of ‘curved lateral border’ and ‘talar head coverage’ are all poorly discriminating factors in walking-age children).

We observe that there is variation in DPC casting protocols in terms of short and long leg casts, how many serial casts are attempted, instructions on weight-bearing, and whether bilateral feet are treated at the same time or sequentially, and how these are related to important contextual factors such as the child’s age, size, mobility, the strength of caregivers (to lift the child), family and socio-economic situation, and schooling. The preferred cast change interval time is usually every one or two weeks. Despite the variations in techniques, in general, practitioners apply the principles of the Ponseti method to sequentially correct the clubfoot deformity as far as possible with long leg serial casting and manipulation. Contextual factors also influence the variability in the use of orthotics and the need for a tibialis anterior tendon transfer (TATT) to the lateral cuneiform, with reasons provided for the use of each according to their availability and perceptions about compliance. For example, patients who travel long distances at great expense would not be able to afford a continued orthotic management regime.

The survey results identify key physiotherapy exercises whilst casting and following the removal of the cast to aid fast and optimal recovery, especially for bilateral clubfoot cases. This is important as children with clubfoot have a specific way of walking that aims to compensate for their condition. They may walk with their knees in hyperextension and their pelvis tilting forward, which causes their lower back to arch. This happens because their centre of gravity has shifted backward, and their feet and ankles do not provide enough push. As a result, their core muscles and foot muscles become weak. After being treated with casts, clubfoot patients not only need to regain their muscle strength, but also work on their core muscles. The survey results underline the importance of a holistic and multidisciplinary approach, including physiotherapy involvement, through all phases of treatment planning, deformity correction, maintenance phase, and rehabilitation.

As discussed in the Introduction, there are some published studies on the outcomes of the application of the Ponseti Principles in older children. We have summarised the key publications from the last 10 years in Table 2 below. Despite the short-term data, relapse rates have been noted to be significantly high, particularly among older children [17,18]. It has often been concluded that this is due to brace compliance, a common problem seen in infant clubfoot treatment. Orthotic use and orthotic brace repair and replacement are all significant challenges in low-income settings where healthcare access is challenging. It is worth noting that children presenting with untreated clubfoot in these settings, especially in countries where infant clubfoot treatment is available, have usually self-selected as having the most challenging socio-economic situations. It is therefore unsurprising that adherence to follow-up is poor. A common theme in these papers is a recommendation for higher quality and higher volume studies. An impressive theme among the studies in Table 2 is that the loss to follow-up rate is very low. Further studies that are funded sufficiently to cover patient travel costs in low-income settings are needed.

In summary, this study’s findings reveal variations in the treatment approaches and practices for older children with clubfoot, while also underscoring important points of consensus. This mirrors the dynamic nature of clubfoot treatment for older children, which continues to evolve through ongoing research and clinical practices. Our results align with the broader literature, emphasising the importance of a multidisciplinary approach in managing clubfoot [22]. While some authors advocate for complex surgical intervention [23], highlighting its potential for swift correction, the prevailing consensus among respondents underscores the crucial role of pre-operative casting to correct the midfoot, which is fully in line with the application of the Ponseti clubfoot principles. The necessity of long-term follow-up is widely acknowledged to monitor for potential relapses or residual deformities [24]. Parental education remains a cornerstone in clubfoot management, as it empowers caregivers with the knowledge and skills needed to actively participate in their child’s treatment and ensures consistent adherence to recommended bracing and follow-up protocols, ultimately contributing to the effectiveness of the chosen treatment plan [25].

These survey findings contribute to the collective knowledge and understanding on the existing practice of multidisciplinary treatment of clubfoot in older children in resource-limited settings. The formative research findings have been reviewed by the study team, project technical advisory group, and project stakeholder group to triangulate the data to influence the design of the content of a new training course on the principles of management for delayed-presenting clubfoot (in walking-age children aged 2–10 years). 

We suggest that further research may be beneficial on areas where there is no consensus and/or limited evidence base, such as rehabilitation exercises in or out of post-op casts, management approaches for recurrence, use of orthotics, whether there is a maximum age for weekly cast changes, criteria and evidence for treating bilateral clubfeet at the same time or sequentially, identifying causes of recurrence and best management options, and documenting adaptive gait disturbances in clubfoot and the efficacy of directed physiotherapy interventions.

As the survey was undertaken in English only, the results are limited in that they do not reflect the experiences of French- or Portuguese-speaking practitioners in Africa. The sample for some of the questions is smaller (e.g., the surgical technique questions that were not applicable to all respondents) which may reduce the generalisability of these results; therefore, we have stated number counts as well as percentages. Selection bias could be present due to the sampling strategy to target known experienced practitioners; however, the cascaded invitation through Global Clubfoot Initiative members sought to provide an opportunity to collect a broader range of experienced practitioners. Interpretation bias could be present as some of the study team and technical advisory group have previously developed and published protocols on the management of DPC using the Ponseti principles; however, we have sought to mitigate this through the inclusion of researchers and focus group participants who had not been involved. 

## 5. Conclusions

The survey findings identify key areas of consensus in the overall principles of management for delayed-presenting clubfoot in walking-age children and the implementation of the Ponseti principles for this patient group. Some differences that exist in the approach may reflect practitioner adaptations to the surgical and rehabilitation resources available in the local context and the socio-economic situation of the patient and the family. Although the technique for DPC management is not yet standardised, the experience of the practitioners surveyed demonstrates how the principles can be well adapted for different geographical and healthcare contexts and tailored to the context of the patient and family, as part of a holistic and flexible approach.

## Figures and Tables

**Table 1:** Summary of main quantitative results from survey of practitioners treating older children with clubfoot.

Question Topic	Answer Selected	Number of Respondents	% Respondents (per Question)
Plaster casting	Long leg casts	41	83.7
	Short leg casts	5	10.2
	Other	3	6.1
	Total number of responses	49	100
Weight-bearing status in serial cast (pre-operative)	No weight-bearing in cast	27	54.0
	Weight-bearing in cast	10	20.0
	It depends	13	26.0
	Total number of responses	50	100.0
Preferred interval between cast changes	One week	24	48.0
	Two weeks	20	40.0
	Other	6	12.0
	Total number of responses	50	100.0
Recommended use of night splints or other orthotics	Yes	42	84.0
	No	6	12.0
	Don’t know	2	4.0
	Total number of responses	50	100.0
Surgical goal of achieving full correction of equinus deformity	Plantigrade	5	13.2
	Dorsiflexion 10 degrees	17	44.7
	Dorsiflexion of 15 degrees or more	12	31.6
	Other	4	10.5
	Total number of responses	38	100.0
Inclusion of posterior ankle capsular release in protocol	Always	3	7.9
	Sometimes	26	68.4
	Never	6	15.8
	Not applicable to me	3	7.9
	Total	38	100.0
Cast wedging to gain further dorsiflexion	Yes	13	35.1
	No	22	59.5
	Not applicable to me	2	5.4
	Total number of responses	37	100.0
Preferred technique for Achilles tendon lengthening	Achilles tendon lengthening	7	18.4
	Tenotomy	9	23.7
	Either tenotomy or lengthening	22	57.9
	Total number of responses	38	100.0
Pre-operative imaging	Yes	18	47.4
	No	20	52.6
	Total number of responses	38	100.0
Anaesthesia used for Achilles tendon lengthening age 2–10 years	Local anaesthesia	5	13.2
	General anaesthesia	25	65.8
	It depends	8	21.0
	Total number of responses	38	100.0
Experience of recurrence or over-correction in your clubfoot cases (multi-answer question)	Yes, recurrence	41	82.0
	Yes, over-correction	6	12.0
	No	7	14.0
	Not applicable	2	4.0
Post-operative casting performed	Yes	26	52.0
	No	19	38.0
	Not applicable	5	10.0
	Total number of responses	50	100.0
Weight-bearing status in cast post-surgery	Weight-bearing as tolerated	10	26.3
	Partial weight-bearing	4	10.5
	Non weight-bearing	24	63.2
	Total number of responses	38	100.0

**Table 2:** Selected delayed-presenting clubfoot studies since 2013. Comparative data are presented on case selection, study design, and relapse rates.

First Author Name	Year of Publication	Number of Feet in the Study	Mean Age (and Range)	Consecutive Patient?	Ponseti Casting Principles Specifically Followed (Y/N)	Follow-Up Period in Years (and Range)	Casting Failure Rate	Loss to Follow-Up Rate	Relapse Rate (%)	Study Type
Ayana [7]	2014	32	4.4 (2–10)	Yes	Yes	3 (2–4)	0	3%	13%	Prospective case series
Bankskota [19]	2013	55	7.4 (5–10)	Yes	Yes	2.6 (2–3.3)	16%	17%	16%	Retrospective case series of patients treated in 1 year
de Podesta Haje [17]	2020	429	3 median (1.6–5.0) IQR	Not mentioned	Yes	1.3 median (0.7–2.5) IQR	13%	24% if over 2 years	31% (in those followed up for over 2 years)	Retrospective case series from 15 centres over 3 years
Faizan [20]	2015	28	2.7 (1–3.5)	Yes	Yes	2.7 (1.5–3.5)	7%	0	14%-repeat tenotomy and tibialis anterior tendon transfer	Prospective study over 23 months
Mehtani [8]	2017	62	3.1 (1.1–12)	Not mentioned	No, cavus corrected simultaneously with the adduction correction	3 (1.2–4)	6%	Follow-up was necessary for included study cases	11%	Prospective two-centre study
Shah [18]	2018	18 untreated cases	3.3 (1–8.4)	Yes	Yes	2.96 (1.2–5)	0	0	44%	Prospective data collection in a registry
Sinha [21]	2016	41	3.02 (1–10.3)	Not mentioned	Yes, but plantar fascia releases were required in some cases as there was persisting cavus deformity following cast protocol	2.6 (2–3.9)	0	0	17%	Prospective case series

## Data Availability

The data that support the findings of this study are available on reasonable request from the corresponding author, G.D. The data are not publicly available due to their containing information that could compromise the privacy of research participants.
